# FATHERS’ PARTICIPATION IN NEONATAL UNITS: AN ONGOING PROCESS

**DOI:** 10.1590/1984-0462/;2019;37;3;00020

**Published:** 2019-10-10

**Authors:** Zeni Carvalho Lamy

**Affiliations:** a Department of Public Health, Graduate Program in Public Health, Universidade Federal do Maranhão, São Luís, MA, Brazil.

For a long time, care for preterm newborn infants admitted to a neonatal unit was restricted only to the hospital staff. Fathers and mothers were visitors that had restricted hours and no autonomy over their child. Over the past few decades, newborns have had a greater chance for survival in neonatal units. As such, the negative impact of restricted parental care on establishing the bond between parents and children and its consequences, such as abandonment, abuse and neglect, has been identified.^1^

Given this evidence, changes have been devised and Neonatal Intensive Care Units (NICUs) around the world have begun to open their doors to parents. However, in effect, this stimulus has mainly centered on mothers’ participation. Historically, men have been excluded from childbirth and even from providing childcare and being a paternal figure, which is strongly linked to family support. However, two interconnected movements have relativized this issue: new family and gender arrangements, with a social demand for contemporary parents to exercise a more implicit and active role in parenting with regard to living with and caring for their children, and changes in health services.^2^

In Brazil in recent years, fathers’ participation has been stimulated. Some examples include the Companion Law, which, although not specific to the father, opened up the possibility of having him present with his wife in pre-delivery, childbirth and the postpartum period;^2^ GM Ordinance No. 930, which regulates neonatal hospitalization in Brazil and included the participation and protagonism of both parents in the care of hospitalized children, guaranteeing them free access and the ability to stay permanently with their child;^3^ the partner’s prenatal preparation, an essential strategy to give attention to pregnancy, childbirth and birth;^4^ paternity leave, which has been re-discussed and extended; and the Kangaroo Method, which includes strategies aimed at the pregnant couple and the effective participation of the father in the child’s hospitalization, including the kangaroo position, which has been shown to be advantageous in improving neonatal outcomes.^5^

When a baby faces neonatal hospitalization, the father, in general, is the first one to ask for news, and the team does not always welcome him and encourage his participation. In this issue of the *Revista Paulista de Pediatria*, Soares et al.^6^ discuss this issue. This important article presents multi-professional teams’ perceptions and demonstrates that there are still disagreements among them. For some, the role of the father is as a provider and not as a caregiver. For others, fathers’ participation should involve putting the child in a kangaroo position, changing diapers, giving the child a bath, and supporting the mother in breastfeeding. Giving voice to these professionals enables reflections on NICU practices and denotes that urgent changes need to take place. Teams committed to good clinical practice need to effectively include fathers in neonatal care as a human right, not just as a benefit for the child and the mother.
